# Sleep-amount differentially affects fear-processing neural circuitry in pediatric anxiety: A preliminary fMRI investigation

**DOI:** 10.3758/s13415-017-0535-7

**Published:** 2017-09-14

**Authors:** Christina O. Carlisi, Kevin Hilbert, Amanda E. Guyer, Monique Ernst

**Affiliations:** 1 Section on Development and Affective Neuroscience, National Institute of Mental Health, National Institutes of Health, 15K North Drive, Bethesda, MD 32541 USA; 2 Institute of Psychiatry, Psychology and Neuroscience, Department of Child and Adolescent Psychiatry, King’s College London, London, UK; 3 Behavioral Epidemiology, Institute of Clinical Psychology and Psychotherapy, Technische Universitat Dresden, Dresden, Germany; 4 Center for Mind and Brain, University of California, Davis, Davis, CA USA

**Keywords:** Anxiety, Sleep, Emotion, FMRI

## Abstract

Insufficient sleep, as well as the incidence of anxiety disorders, both peak during adolescence. While both conditions present perturbations in fear-processing-related neurocircuitry, it is unknown whether these neurofunctional alterations directly link anxiety and compromised sleep in adolescents. Fourteen anxious adolescents (AAs) and 19 healthy adolescents (HAs) were compared on a measure of sleep amount and neural responses to negatively valenced faces during fMRI. Group differences in neural response to negative faces emerged in the dorsal anterior cingulate cortex (dACC) and the hippocampus. In both regions, correlation of sleep amount with BOLD activation was positive in AAs, but negative in HAs. Follow-up psychophysiological interaction (PPI) analyses indicated positive connectivity between dACC and dorsomedial prefrontal cortex, and between hippocampus and insula. This connectivity was correlated negatively with sleep amount in AAs, but positively in HAs. In conclusion, the presence of clinical anxiety modulated the effects of sleep-amount on neural reactivity to negative faces differently among this group of adolescents, which may contribute to different clinical significance and outcomes of sleep disturbances in healthy adolescents and patients with anxiety disorders.

**Electronic supplementary material:**

The online version of this article (10.3758/s13415-017-0535-7) contains supplementary material, which is available to authorized users.

## Introduction

Adolescence is a period of heightened risk for the onset of psychiatric problems, particularly anxiety (Beesdo, Knappe, & Pine, 2009; Pine, Cohen, Johnson, & Brook, 2002; Pine & Fox, 2015). Moreover, adolescents show high levels of objectively measured sleepiness and changes in mood and affect regulation in response to sleep loss (Gregory & Sadeh, 2012). Disrupted sleep quantity has been shown to have a wide range of negative consequences on health, well-being, and motor and cognitive function (Orzeł-Gryglewska, 2010). For example, chronic sleep loss has been linked to diabetes/obesity (Knutson, Spiegel, Penev, & Van Cauter, 2007), poor academic functioning, drug use problems, and low mood (Roberts, Roberts, & Duong, 2009). In the laboratory, sleep-deprivation manipulation has been shown to decrease positive affect and increase anxiety symptoms (Talbot, McGlinchey, Kaplan, Dahl, & Harvey, 2010), and decrease vocal expression of emotion, particularly in adolescents (Mcglinchey et al., 2011). Because of our specific interest in anxiety during adolescence, the present work was conducted in healthy and anxious adolescents and was restricted to the effects of sleep amount on emotion processing, and particularly on the neural circuitry underlying emotion processing. Four lines of evidence guided our hypotheses: (1) sleep impacts emotion regulation; (2) emotion circuitry in adolescence is perturbed in (3) anxiety and (4) in the context of sleep disturbances.

### Sleep and emotion regulation

A large literature indicates that sleep loss negatively impacts one’s ability to regulate emotional responses to negative stimuli (Chorney, Detweiler, Morris, & Kuhn, 2008; Czeisler, 2011; Gregory & Sadeh, 2012; Morrison, McGee, & Stanton, 1992; Reid, Hong, & Wade, 2009; Riemann & Voderholzer, 2003; Stein, Mendelsohn, Obermeyer, Amromin, & Benca, 2001; Tkachenko et al., 2014), especially in adolescence (Soffer-Dudek, Sadeh, Dahl, & Rosenblat-Stein, 2011). Furthermore, disruption of the neural circuits underlying fear processing and emotion regulation has been shown both in adolescents with anxiety disorders (Beesdo, Lau, et al., 2009; Mcclure et al., 2007; Monk et al., 2006) and as a function of sleep quality in healthy adolescents (Holm et al., 2009). Lastly, the relationship between emotion regulation difficulties and the presence of anxiety symptoms in young adults is potentiated in cases where individuals also report poor sleep quality (Markarian, Pickett, Deveson, & Kanona, 2013). This latter observation suggests that sleep may play a role in poor emotion regulation in individuals who are already vulnerable to emotional difficulties (Baglioni, Spiegelhalder, Lombardo, & Riemann, 2010; Gruber & Cassoff, 2014). Therefore, compromised sleep might be reciprocally linked to disrupted neural processing of negative emotional stimuli in anxious youth. The present study tests this hypothesis by examining how sleep amount can affect neural responses to negative emotional stimuli differently in clinically anxious versus healthy adolescents.

### Emotion neurocircuitry

In healthy individuals, emotional stimuli are processed via distributed frontolimbic brain networks encompassing four key regions: hippocampus, amygdala, insula, and prefrontal cortex (PFC) (Etkin, Egner, & Kalisch, 2011; Hartley & Phelps, 2010; Milad et al., 2007; Ochsner et al., 2004; Phelps, 2004; Shin & Liberzon, 2009). Within the PFC, the areas implicated in emotion regulation lie in the medial PFC (mPFC), including ventromedial PFC (vmPFC) (Morgan, Romanski, & LeDoux, 1993; Quirk & Beer, 2006; Quirk, Likhtik, Pelletier, & Paré, 2003), dorsomedial PFC (dmPFC) and dorsal anterior cingulate cortex (dACC) (Etkin et al., 2011; Ochsner et al., 2004). The dACC and more rostral vmPFC regions contribute to the appraisal and regulation of fear response (Arnsten & Rubia, 2012; Diekhof, Geier, Falkai, & Gruber, 2011; Etkin et al., 2011; Maier et al., 2012; Quirk & Beer, 2006; Schiller & Delgado, 2010) and work in concert with the amygdala and hippocampus (Milad et al., 2007). The hippocampus modulates amygdala activation to emotional stimuli (Phelps, 2004), and the insula integrates somatosensory information with emotional valence via extensive insula-limbic networks (Adolphs, 2003; Phillips, Drevets, Rauch, & Lane, 2003). The insula is also involved in judgment of emotional facial expressions (Gorno-Tempini et al., 2001; Phan, Wager, Taylor, & Liberzon, 2002). Based on the influence of sleep perturbations on emotional expression and regulation in adolescents (Gregory & Sadeh, 2012; Reid et al., 2009), and the association of anxiety with sleep disturbances (Chorney et al., 2008), the functional integrity of these brain regions is likely to be affected by variability in sleep amount. This link may be exacerbated in clinical anxiety, particularly during the sensitive developmental period of adolescence.

### Anxiety-related neurocircuitry

In patients with an anxiety disorder, perturbations in the aforementioned distributed frontolimbic brain networks are commonly reported (Gilbertson et al., 2002; Etkin & Wager, 2007). In the wider context of emotion processing abnormalities, both adults and children with anxiety have shown biases specifically toward threatening or aversive faces (e.g., fearful, angry; Blair et al., 2008; Robinson, Vytal, Cornwell, & Grillon, 2013; Roy et al., 2008). Activation of the hippocampus, amygdala and insula to negative emotions is potentiated in anxious adults (Etkin & Wager, 2007; Hattingh et al., 2013; Kalisch & Gerlicher, 2014; Stein, Simmons, Feinstein, & Paulus, 2007) and anxious adolescents (Fox & Kalin, 2014). Regarding the PFC, anxious versus nonanxious individuals exhibit top-down hyperresponsiveness of mPFC, including dACC/dmPFC, during conscious appraisal of fear (Vogt, 2005). In contrast, rostral and ventromedial prefrontal regions, which send inhibitory projections to the amygdala (Morgan et al., 1993; Quirk & Beer, 2006; Quirk et al., 2003), are hypoactive in anxious compared to non-anxious individuals, suggesting diminished modulation of fear responses (Greenberg, Carlson, Cha, Hajcak, & Mujica-Parodi, 2013). Finally, reduced (Hahn et al., 2011; Krain et al., 2013), but also stronger (Guyer, Lau, et al., 2008; Krain et al., 2013), limbic-prefrontal connectivity in anxious versus healthy adolescents has been documented. Therefore, these neural mechanisms may all be affected by reduced sleep amount.

### Sleep-perturbations impact on brain function

Sleep disturbances have been associated with alterations in subcortical and mPFC circuits, described above as key structures underlying emotion processing and showing abnormalities in pathological anxiety. These sleep-related neural effects have been proposed as a possible mechanism underlying the relationship between abnormal sleep regulation and emotional difficulties (Goldstein & Walker, 2014; Gruber & Cassoff, 2014; Reidy, Hamann, Inman, Johnson, & Brennan, 2016). Dysregulation of top-down inhibitory control of prefrontal regions over limbic structures has been associated with sleep deprivation, leading to abnormal emotional response (Gujar, Yoo, Hu, & Walker, 2011; Kaufmann et al., 2016; Walker, 2009). Moreover, altered function in dmPFC regions, along with the hippocampus, amygdala, ACC, and insula, has been linked to poor sleep quality (Klumpp et al., 2017; Minkel et al., 2012) and insomnia, which often co-occur with anxiety disorders (Koenigs, Holliday, Solomon, & Grafman, 2010). The typical increase of hippocampal activity during emotion processing is negatively impacted by sleep deprivation in healthy individuals (van Der Helm & Walker, 2009; Yoo, Hu, Gujar, Jolesz, & Walker, 2007). Lastly, the hippocampus and dACC are important components of the default mode network (DMN), a proposed network that is active at rest but down-regulated during task-based cognitive engagement. Of note, the DMN is involved in worry and rumination, particularly in the context of anxiety (Buckner, Andrews-Hanna, & Schacter, 2008; Raichle et al., 2001). Sleep deprivation has been suggested to induce abnormal DMN activation or to negatively impact effective allocation of brain resources to task-relevant demands in a way that may explain enhanced emotionality associated with sleep deprivation (Regen et al., 2016).

Based on this background, this study assessed whether the presence of an anxiety disorder moderates sleep effects on emotion processing in adolescents. The sleep measure consisted of a retrospective self-report on sleep amount accumulated in the past 3 nights preceding the study. Three hypotheses guided this work: (1) vmPFC activation would be reduced in anxious relative to healthy adolescents during negative emotion processing; (2) sleep amount would have a stronger negative impact on the neural correlates of emotion processing, including hippocampus, amygdala, insula, and mPFC in anxious versus healthy adolescents; and (3) the functional connectivity of these regions would be influenced by sleep differently in anxious compared to healthy adolescents, yet specific predictions are limited due to a lack of existing data to drive expectations.

## Method

### Participants

Participants were 33 adolescents (11 males; mean age 14.1 years), 14 patients with a DSM-IV Axis I anxiety disorder (American Psychiatric Association, 2000) and 19 healthy adolescents (HA). All patients were clinically diagnosed with at least one anxiety disorder, as evaluated by a senior clinician using the semistructured Kiddie Schedule for Affective Disorders and Schizophrenia, present and lifetime (K-SADS-PL; Kaufman et al., 1997). Four patients had more than one comorbid anxiety disorder. Six anxious patients had comorbid secondary depression in addition to a primary anxiety disorder. All anxious adolescents (AA) were medication-free and seeking outpatient psychiatric treatment through the National Institute of Mental Health (NIMH). Physical and mental health of all participants was evaluated through physical examination by a physician or nurse practitioner and a clinical interview with a psychiatrist or clinical psychologist. Exclusion criteria included current Tourette’s syndrome, obsessive-compulsive disorder, posttraumatic stress disorder, conduct disorder, exposure to extreme trauma, or suicidal ideation; lifetime history of mania, psychosis, pervasive developmental disorder, or clinically significant disruptions in sleep patterns; current psychiatric medication exposure; structural abnormalities on clinical MRI scan or safety/data quality MRI exclusion criteria; or IQ less than 70.

Healthy controls had no lifetime diagnosis of any psychiatric or neurological condition and no first-degree relative with a mood disorder. All participants were recruited from the community by advertisements and word of mouth. The study was approved by the NIMH Institutional Review Board. All participants and legal guardians provided written informed assent and consent and were compensated for their time.

### Assessment tools

IQ was measured using the Vocabulary and Matrix Reasoning subscales of the Wechsler Abbreviated Scale of Intelligence (Wechsler, 1999). Socioeconomic status (SES) was obtained through parental report and was calculated based on the Hollingshead’s index of social position for education and occupation categories (Hollingshead, 1975). Adolescents and parents completed the Screen for Child Anxiety Related Emotional Disorders, parent and child versions (SCARED-p/c) to assess trait-based anxiety symptoms (Birmaher et al., 1997). Additionally, adolescents completed the Childhood Depression Inventory (CDI; Kovacs, 1978) and the State-Trait Anxiety Inventory (STAI), trait version (Spielberger, Gorsuch, Lushene, Vagg, & Jacobs, 1983).

### Sleep questionnaire

The sleep questionnaire was developed in the lab to provide two types of self-reported measures, sleep amount and tiredness, and was administered to participants taking part in a larger fMRI study in which sleep amount was not a primary focus. To measure sleep amount, subjects reported how many hours they slept each of the 3 nights preceding the scan. Specifically, they were asked to indicate the time of sleep onset and waking for each of these three nights. Based on this information, average sleep amount for 3 three nights was computed and rounded up to the nearest minute. For tiredness, subjects rated on a scale of 1 (*not tired at all*) to 10 (*extremely tired*) how tired they felt just prior to entering the scanner. Participants were administered the sleep questionnaire right before scanning.

### FMRI task

We probed neural response during the viewing of negative facial expressions, without constraining attention to any particular subjective state. To this aim, the face-attention paradigm (Guyer, Monk, et al.,^2008^) was used, with a focus on negative versus neutral faces, across attention states.

**Fig. 1:**
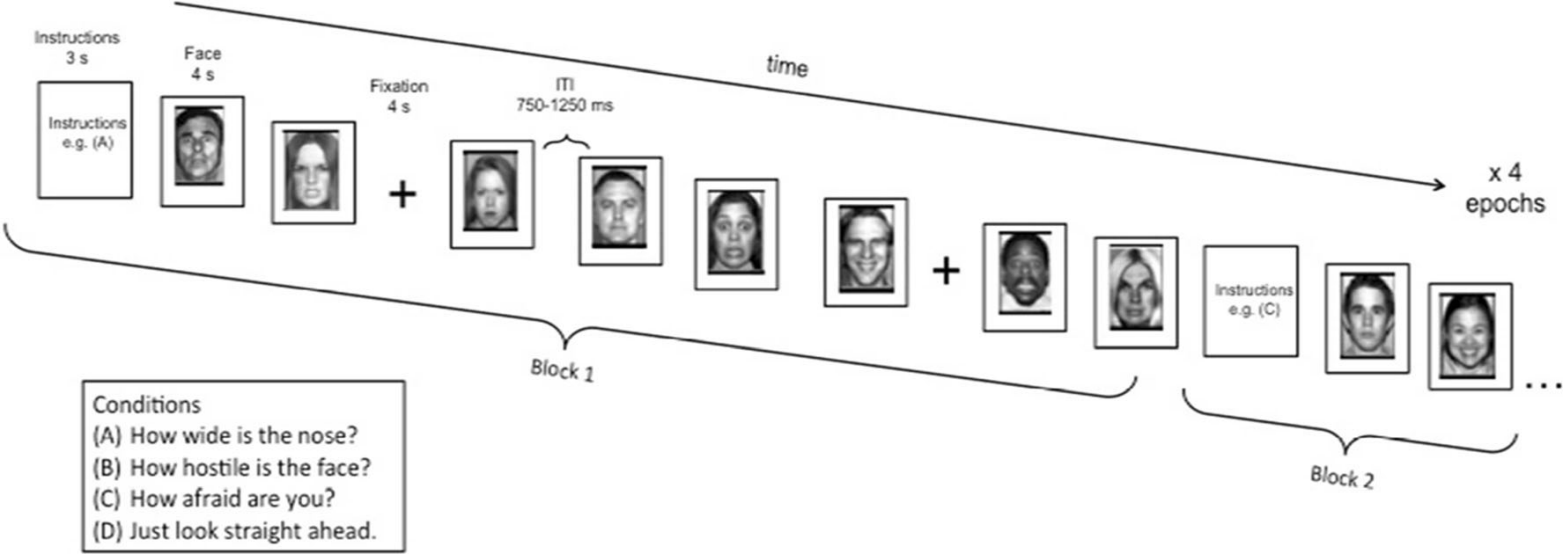
Task design and sample stimuli. The task consisted of four epochs containing four blocks each. Each block consisted of 10 trials, eight neutral or emotional faces and two fixations. Order of neutral and emotional faces and fixation crosses varied on a random trial-by-trial basis. At the start of each separate block, participants were instructed to rate on a 1-to-5 Likert scale the nose width, hostility intensity, or fear intensity of each face. Subjects responded to the same question for an entire block. There was also one block per epoch of passive viewing where subjects were not asked to make a response. Block order was randomized across epochs and across subjects. ITI = intertrial-interval. (Adapted with permission from Guyer et al. 2008)

The task consisted of a single 160-trial run divided into four epochs, with four blocks of 10 trials for each epoch (eight faces, two of each emotion, and two fixation trials; see Fig. 1). The task had participants view and rate neutral and emotional faces (angry, fearful, and happy), attending to their subjective emotional reactions to feelings of hostility or fear, or to a nonemotional physical feature of the faces, or during passive viewing of faces. Thus, the attention blocks represented four different attention states per instructions: “Just look at the face”; “How afraid are you?”; “How hostile is the face?”; and “How wide is the nose?”. However, given the study hypotheses, effects of the attention manipulation were not analyzed.

For each block, instructions were presented for 3 s at the onset of the block, and each face was presented for 4 s, during which the subject was required to make a response or passively view the face, depending on instructions. Additionally, within each block, two fixation trials were randomly presented between face stimuli for 4 s. Following each face or fixation, intertrial interval (ITI) varied between 750 and 1,250 ms. Blocks were randomized across participants. To respond to the three rating questions, participants used a five-button response box (Waukesha, WI) providing a 5-point scale ranging from 1 (*not at all*) to 5 (*extremely*). Task stimuli consisted of 56 actors taken from three widely known face-stimulus sets (Ekman & Friesen, 1976; Gur et al., 2001; Tottenham, Borscheid, Ellertsen, Marcus, & Nelson, 2002) presenting four facial expressions (happy, angry, fearful, and neutral). Stimuli were randomly selected so that each participant viewed 32 different actors during the task. Each actor was randomly selected to portray the same emotion across the entire task for a given participant (e.g., a given actor might be randomly selected to portray “fear” for one participant, while that same actor portrayed “anger” for another participant, thus allowing us to control for variability of nonemotional features in the faces (for additional details, see Guyer, Monk, et al., 2008).

Task performance (mean reaction time; MRT) for blocks where subjects were asked to make a response was analyzed using nonparametric repeated-measures analysis of variance (rANOVAs), with diagnostic group as the between-subjects factor and face emotion as the within-subjects factor.

Data analyzed in the present study were collected as part of a larger fMRI investigation, which administered the sleep questionnaire as part of a battery of prescan screening measures; data from healthy adolescents and patients during other conditions of this task have been published elsewhere (Beesdo, Lau, et al., 2009; Guyer, Monk, et al., 2008; Hariri, 2009; Lau et al., 2008), and the primary goal of the larger study was not to investigate the impact of sleep on brain function. These measures of sleep amount and tiredness were administered right before scanning. The present study should be considered a preliminary investigation into the possible effects of sleep on negative emotion processing.

### Behavioral data analysis

All statistical analyses were conducted in JASP (Version 8.5; https://jasp-stats.org/) using Bayesian analysis based on posterior probabilities rather than frequentist *p* values, which rely on the sampling intentions of the investigator. Models were favored if BF_10_ > 10, indicating strong evidence for the tested model over the null hypothesis. In instances where BF_10_ was sufficiently large (>1,000), Log(BF_10_) is reported, where values >1 indicate strong evidence for the model. For clarity, where appropriate, we also report null-hypothesis significance tests (NHST), including *p* values. For results of task performance as assessed by MRT, see Supplementary Table S1.

### Imaging data

Imaging data were acquired on a General Electric Signa 3-Tesla scanner, and analyzed using the Statistical Parametric Mapping Software package (SPM8; University College London). An echo-planar single shot gradient echo T2*-weighted sequence was used to collect 23 axial slices of 5-mm thickness, parallel to the AC-PC-line (repetition time = 2,000 ms; echo time = 40 ms; field of view = 240 mm; 64 × 64 matrix, 3.75 × 3.75 mm voxels). The anatomical scan used a magnetization prepared gradient echo (MPRAGE) sequence to collect 180 1 mm sagittal slices (field of view = 256 mm; repetition time = 11.4 ms; echo time = 4.4 ms; matrix = 256 × 256; inversion time = 300 ms). Data were analyzed for subjects who successfully completed the task and stayed within 3.0 mm of motion in any plane.

#### Individual-level fMRI analysis

Preprocessing procedures comprised correction for slice timing and motion, coregistration to anatomical scans, normalization to a Montreal Neurologic Institute (MNI) T1-weighted template, image reslicing to an isotonic resolution of 2 × 2 × 2 mm, and smoothing with an 8 mm full-width half-maximum (FWHM) Gaussian kernel. Event-related blood-oxygen-level dependent (BOLD) responses were estimated for each subject at an individual level. Subjects did not differ on motion parameters: head translation, *U*(31) = 157, *z* = 0.87, *p* = .40, BF_10_ = 0.7, and rotation, *U*(31) = 108, *z* = 27.45, *p* = .38, BF_10_ = 0.4, within 3-D Euclidean space or on rotation or translation within any one plane. Therefore, motion parameters were not included in the model. Sixteen conditions of interest (four emotions by four attention states) and one condition of no interest (onset of instructions) were modeled using the general linear model (GLM). Based on findings from McClure et al. (2007) that showed similar neural responses to fearful and angry faces, the fearful and angry face trials were pooled together to probe the effects of negative emotion in general. Thus, the contrast of negative (angry and fearful) versus neutral faces collapsed across all attention conditions was the contrast of interest that was brought to the group-level comparison.

#### Group-level fMRI analysis

A full-factorial design matrix was used to investigate the interaction of group by sleep amount and the main effect of group on BOLD activation to negative faces minus neutral faces. The main effect of sleep amount across the whole brain was additionally examined via regression analysis. Happy faces were not included in the models, based on the fact that fear representation and regulation of emotional response to negative cues is the most salient process for anxiety. A cluster-size-based threshold, computed via a Monte Carlo simulation (Slotnick, Moo, Segal, & Hart, 2003; Slotnick & Schacter, 2004), was used to determine statistical significance at the group level and to correct for multiple comparisons. For calculating the minimum cluster size, 5,000 iterations based on the matrix, slice number, smoothing kernel, and voxel size were carried out with a Type I error of *p* < .005 in the individual voxels. Accordingly, a minimum cluster size of k = 150 voxels was required for a corrected cluster-wise threshold of *p* < .05. In addition, to better understand the nature of the Group × Sleep interactions, individual mean activity via the first eigenvariate were extracted from the significantly activated clusters in the Group × Sleep interaction contrast. These extracted values were then explored via regression analyses in JASP to decompose the direction of statistical interactions.

#### PPI analysis

To further characterize the regions sensitive to the Group × Sleep Amount interaction, a psychophysiological interaction (PPI) analysis was conducted across the whole brain using, as seeds, the peak activation of clusters in the dACC and hippocampus (located at MNI (x, y, z): 2, 6, 26, and −20, −20, −18) activated by the interaction. The gPPI toolbox (Mclaren, Ries, Xu, & Johnson, 2012) was used for these analyses. Individual PPI connectivity analyses were conducted at the individual level and entered into group analyses. For these group analyses, separate full-factorial models included sleep amount as a covariate. In line with earlier studies from our group (Monk et al., 2008), the PPI statistical threshold was set at *p* < .005 with a cluster threshold of k > 20 voxels.

## Results

### Demographics and anxiety ratings

Descriptive statistics showed that groups were well - matched on demographic factors (see Table 1). As expected, anxiety ratings were significantly higher in the AA group compared to HA group.

**Table 1:** Sample characteristics

Demographics, mean (*SD*)	Healthy adolescents (*N* = 19)	Anxious adolescents (*N* = 14)	Test statistic (Mann–Whitney *U)*	*p* value	BF_10_
Age, years	14.16 (2.37)	14.05 (2.16)	128.00	.872	0.34
Gender, M/F	7/12	4/10	–	–	0.44
IQ	107.58 (15.33)	107.02 (13.34)	138.50	.843	0.34
SES	60.26 (23.44)	54.43 (28.56)	117.00	.577	0.39
Diagnoses (*N*)					
GAD		4			
SocAnx		3			
SepAnx		3			
GAD + SocAnx		3			
SocAnx + SepAnx		1			
MDD		6			
Anxiety ratings					
CDI	3.95 (4.29)	12.21 (9.48)	214.50	.002	17.67
SCARED-child	11.92 (7.43)	32.00 (14.89)	244.50	<.001	LogBF_10_ = 6.93
SCARED-parent	4.07 (3.94)	31.93 (13.01)	264.00	< .001	LogBF_10_ = 16.27
STAI-trait	28.18 (7.02)	41.29 (10.12)	226.00	<.001	181.36
Sleep measures					
Hours slept	8.51 (1.13)	7.77 (1.36)	105.50	.321	1.01
How tired	4.71 (1.61)	5.00 (2.42)	147.50	.602	0.34

*Note.* GAD = generalized anxiety disorder; SocAnx = social anxiety disorder; SepAnx = separation anxiety disorder; MDD = major depressive disorder; CDI = Childhood Depression Inventory; SCARED = Self-Report for Childhood Anxiety Related Disorders (child/parent version); STAI = State-Trait Anxiety Inventory

### Sleep measures

No group differences were observed in sleep amount or tiredness (see Table 1; Supplementary Fig. S1). Sleep amount across both groups was between 5 and 12 hours (HAs = 6–12, AAs = 5–10). Across both groups, sleep amount was negatively correlated with tiredness (Spearman’s *r* = −.51, BF_10_ = 5.1, *p*= .002), suggesting that, as expected, the less sleep subjects reported, the more tired they felt. When examined within each group separately, these variables were not correlated. Moreover, using Fisher’s *r*-to-*Z* transformation, the correlations within each group were not statistically different from one another (*Z* = 0.31, *p* = .38). Across groups, STAI-C trait anxiety ratings correlated moderately with tiredness (Spearman’s *r* = .36, BF_10_ = 2.5, *p* < .05). SCARED-c/p or CDI measures did not correlate with sleep measures across both groups or in each group separately.

### Imaging results

#### Whole-brain task-related activation maps

Group maps of differences in activation (negative vs. neutral faces) across the whole brain are summarized below (see Table 2a). Figures are presented in the Supplement (Fig. S2). Three clusters were differentially activated in the AA versus HA group. Two clusters, in right and left cerebellum, were more activated in the AA relative to the HA group. Another cluster, centered in the vmPFC, was less activated in AAs compared to HAs.

**Table 2:** Differential activation patterns in the whole-brain analysis

Contrast	Region	Side	Voxels	*t*	*x*	*y*	*z*
(a) Group effect on whole-brain activation							
AA > HA							
Cerebellum		R	702	5.24	14	−50	−26
Cerebellum		L	213	3.84	−22	−62	−28
HA > AA							
Ventromedial prefrontal cortex (BA 24/32)		B	279	3.88	8	34	8
(b) Group effect on whole-brain activation × sleep amount							
(AA × sleep) > (HA × sleep)							
Anterior cingulate cortex (BA 24/33)		B	308	4.13	2	6	26
Hippocampus/amygdala/occipital^1^		L	319	3.94	−12	−40	−6
Middle temporal gyrus		L	210	3.77	−54	−62	14
Lingual gyrus/cerebellum		R	150	3.31	10	−52	−6
(HA × sleep) > (AA × sleep)							
No differential activation							

*Note.* Voxels = number of voxels per cluster; *x, y, z* = MNI coordinates of peak voxel; AA = anxious adolescents; HA = healthy adolescents; R = right side; L = left side; B = bilateral; *p* < .05, corrected

^1^This cluster extended to several areas. Given our a priori interest in the hippocampus, the hippocampus subpeak within the overall cluster (−20, −20, −18) was used as the PPI seed

#### Effects of sleep amount on brain activation to negative versus neutral faces

**Fig. 2:**
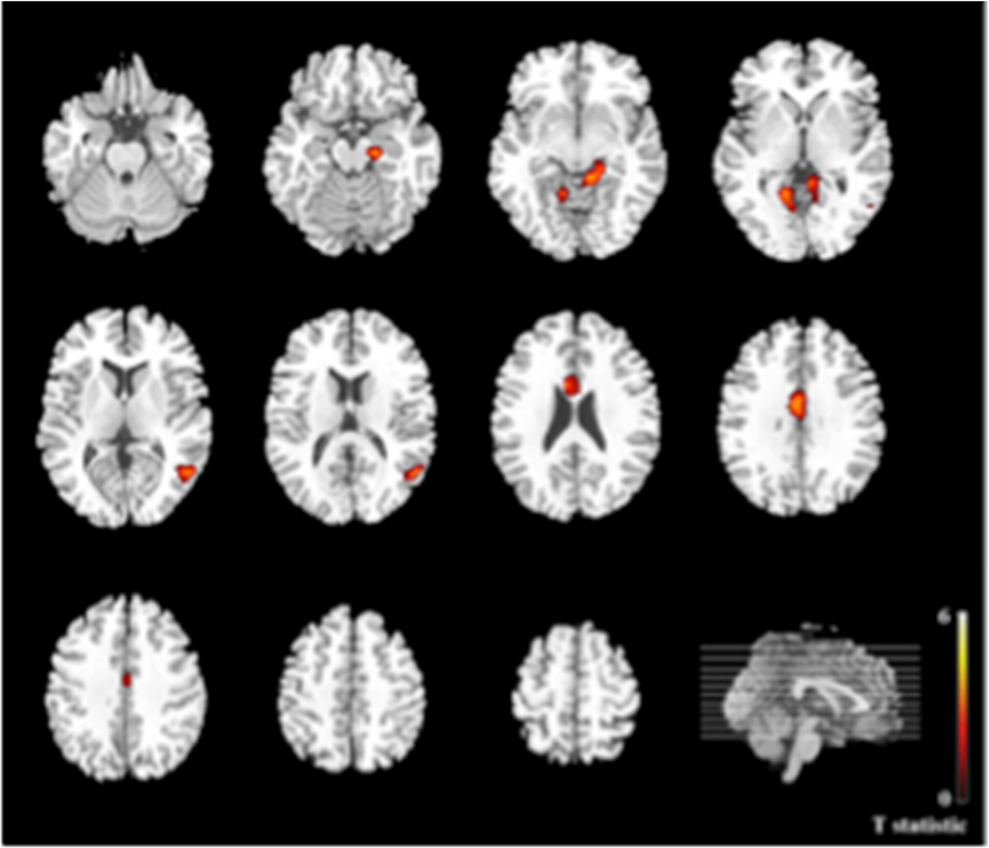
Whole-brain between-group activation differences for negative > neutral faces covaried with sleep amount; Anxious Adolescents > Healthy Adolescents × Sleep Amount. Axial brain slices depicting whole-brain activation differences between anxious adolescents relative to healthy adolescents. The right side of the image corresponds to the right side of the brain, *p* < .05, corrected. (Color figure online). Anxious Adolescents > Healthy Adolescents × Sleep Amount

For completeness, the covariance maps of whole-brain activation (negative vs. neutral faces) with sleep amount are presented for the whole sample and for each group separately in the Supplement (Fig. S3). Our analyses of interest, that is, the group comparison of these covariance maps (anxiety vs. healthy group), are summarized in Table 2b and Fig. 2. These between-group analyses revealed four significant clusters, all showing greater positive correlation with sleep amount in AAs compared to HAs. These clusters included the dACC, hippocampus extending into amygdala and occipital lobe, middle temporal gyrus, and lingual gyrus extending into cerebellum.

**Fig. 3:**
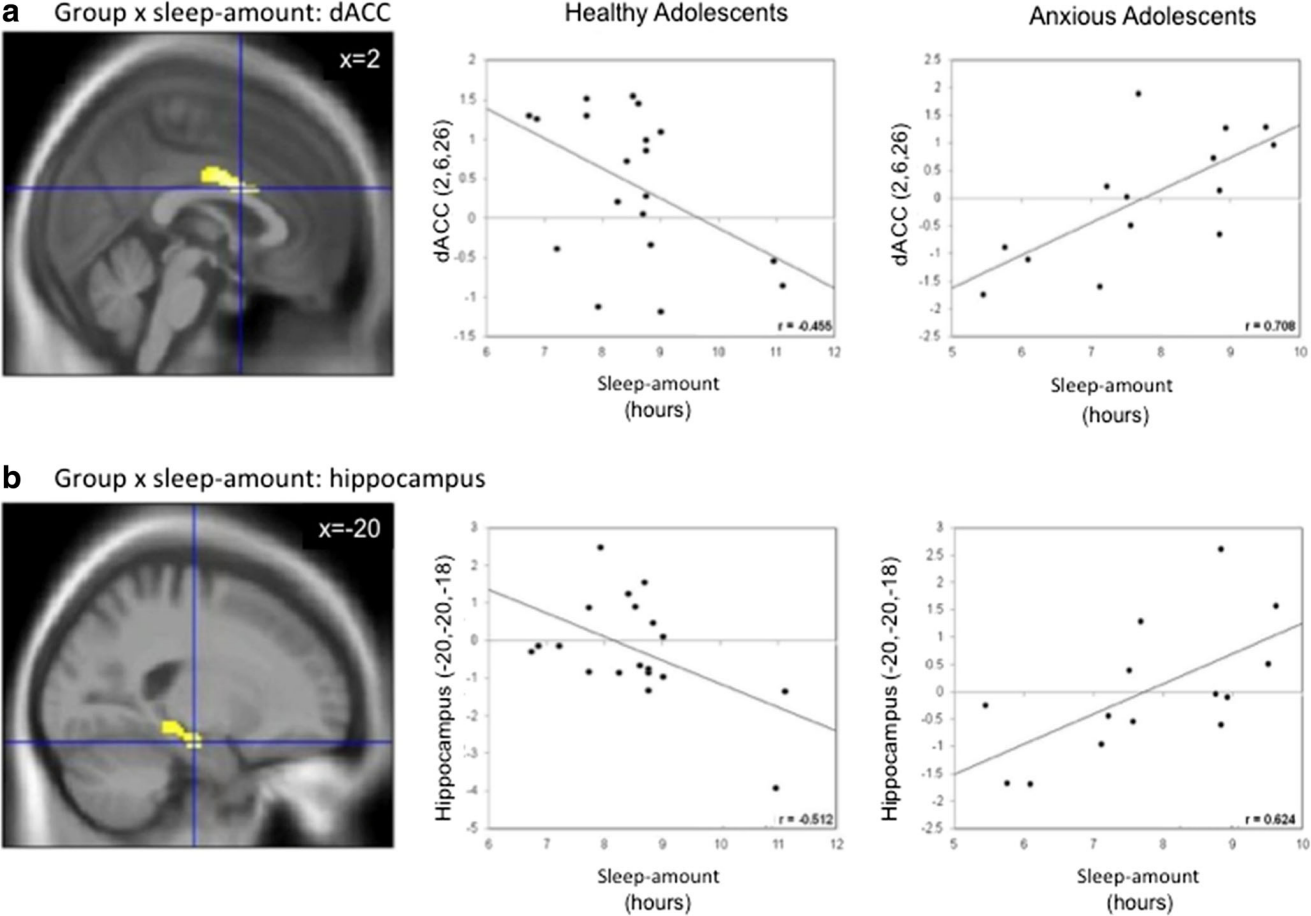
Results of Group × Sleep interaction in ROI extraction of BOLD responses to negative > neutral faces. Also depicted for illustration are the correlations of extracted beta values of brain activation with sleep amount. **a** Sagittal slice showing dorsal anterior cingulate (dACC) ROI × Sleep Amount interaction. **b** Sagittal slice showing hippocampus ROI × Sleep Amount interaction. Coordinates are in MNI space. (Color figure online)

Additional explanatory analyses of the two a priori regions of interest (ROIs), the hippocampus and dACC, were conducted in JASP to plot and determine the direction of effects. For each subject, mean beta values were extracted from 6 mm spheres centered on the peak activation of the dACC, MNI(x, y, z) = 2, 6, 26, and hippocampus, MNI(x, y, z) = −12, −40, −6) clusters. Correlation analyses conducted in JASP between these beta values and sleep amount scores (see Fig. 3) showed similar patterns as those obtained in whole-brain regression analyses. More specifically, these analyses revealed that the correlation between activation and sleep amount was positive in AAs (dACC: *r* = .708, *p* < .01; hippocampus: *r* = .624 , *p* < .05), but negative in HAs (dACC: *r*= −.455, *p* = .05; hippocampus: *r* = −.512, *p* < .05; see Fig. 3). Of note, including CDI scores to account for possible effects of depression symptoms did not affect results.

#### PPI maps

PPI analyses were conducted on dACC and hippocampus seeds, which exhibited significant group differences in their sensitivity to sleep amount when responding to negative versus neutral faces (as shown in Table 2b).

#### Group comparison: dACC PPI

Group differences in whole-brain PPI analysis of dACC revealed six clusters: right insula, bilateral supramarginal gyrus, left dorsolateral PFC (DLPFC), left middle temporal gyrus, and left precentral gyrus. All six clusters showed higher coupling in AAs than in HAs (see Table 3a).

**Table 3:** Differential PPI connectivity patterns with the dorsal anterior cingulate cortex

Contrast	Region	Side	Voxels	*t*	*x*	*y*	*z*
(a) Group effect on dACC PPI							
AA > HA							
Insula		R	43	4.23	42	2	−6
Supramarginal gyrus		R	97	3.97	62	−50	26
Dorsolateral PFC		L	124	3.71	−36	−4	38
Middle temporal gyrus		L	21	3.55	−50	2	−20
Precentral gyrus		L	69	3.41	−48	−4	22
Supramarginal gyrus		L	31	3.19	−56	−24	24
HA > AA							
No differential connectivity							
(b) Group effect on dACC PPI × Sleep Amount							
(AA × covariate) > (HA × covariate)							
Parietal operculum		L	38	3.62	−50	−16	14
Superior temporal gyrus		L	50	3.39	−54	−32	12
(HA × covariate) > (AA × covariate)							
Postcentral gyrus		L	57	3.39	−36	−28	52
Dorsomedial PFC		R	34	3.34	16	22	46
Cerebellum		R	30	3.22	14	−46	−28

*Note.* Voxels = number of voxels per cluster; *x, y, z =* MNI coordinates of peak voxel; dACC: dorsal anterior cingulate cortex; AA = anxious adolescents; HA = healthy adolescents; R = right side; L = left side; PFC = prefrontal cortex; *p* < .05, corrected

Group differences in whole-brain regression analyses of PPI × sleep amount identified five significant clusters (see Table 3b). Coupling between the dACC and two of these clusters, left parietal operculum and left superior temporal gyrus, showed stronger correlations with sleep amount in AAs than in HAs. Coupling of dACC with the other three clusters, left postcentral gyrus, right dmPFC, and cerebellum, showed lower correlations with sleep amount in AAs compared to HAs.

**Fig. 4:**
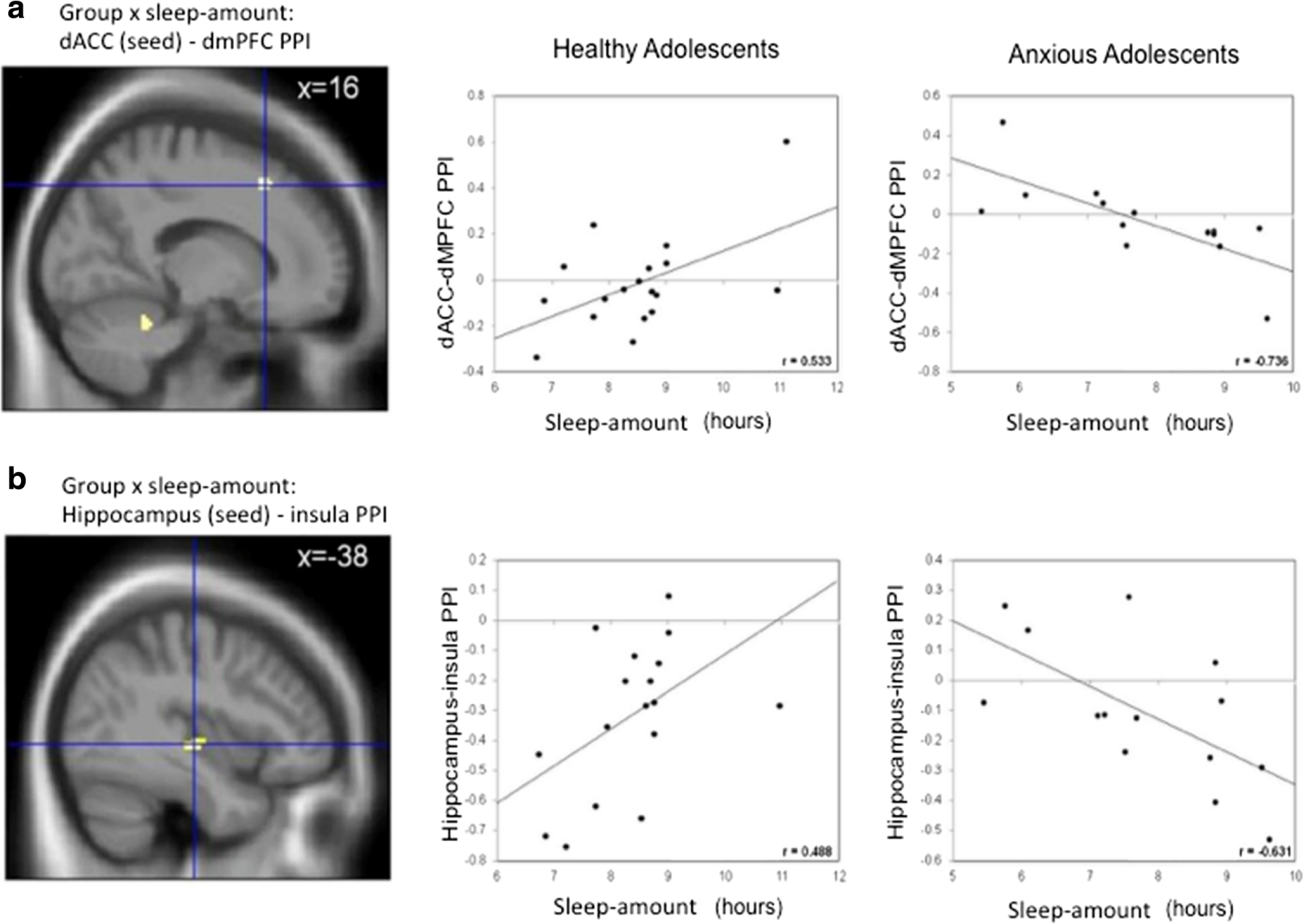
Results of the whole-brain PPI analyses showing group × sleep interaction for [negative > neutral] faces with **a) ** dorsal anterior cingulate cortex (dACC) seed connectivity with dorsomedial prefrontal cortex (dmPFC) and **b) ** hippocampus seed connectivity with insula. Coordinates are in MNI space

Based on the dmPFC’s role in emotion appraisal and fear expression (Etkin et al., 2011; Phan et al., 2002), and to limit the number of tests, mean PPI connectivity estimates between the dACC and dmPFC (MNI: 16, 22, 46; see Table 3b) were extracted to examine the modulation of connectivity between these regions by sleep amount in each group. The analyses revealed a correlation between dACC–dmPFC connectivity and sleep amount that was negative in AAs (*r* = −.736, *p* < .01), and positive in HAs (*r* = .533, *p* < .05; see Fig. 4a).

#### Group comparison: Hippocampus PPI

Group differences in the whole-brain PPI analysis of left hippocampus revealed 11 significant clusters (see Table 4a). Stronger connectivity in AAs compared to HAs was found in four regions, including bilateral insula, left parietal operculum, and left inferior frontal gyrus. Weaker coupling in patients versus controls was seen in six regions, left DLPFC, right supramarginal gyrus, bilateral precuneus, right angular gyrus, and right thalamus.

**Table 4:** Differential connectivity patterns of the hippocampus in the whole-brain analysis

Contrast	Region	Side	Voxels	*t*	*x*	*y*	*z*
(a) Group effect on hippocampus PPI							
AA > HA							
Insula		R	66	3.97	38	−12	−4
Inferior frontal gyrus		L	65	3.45	−26	34	−6
Insula		L	109	3.43	−38	−10	6
Insula		L	50	3.16	−38	6	6
Inferior frontal gyrus		L	23	2.92	−38	32	6
HA > AA							
Precuneus		L	316	4.75	−8	−42	6
Angular gyrus		R	95	4.26	36	−70	42
Precuneus		R	73	3.64	2	−70	40
Dorsolateral PFC		L	65	3.55	−36	6	52
Thalamus		R	22	3.12	12	−32	8
Supramarginal gyrus		R	27	3.07	56	−44	42
(b) Group effect on hippocampus PPI × Sleep Amount							
(AA × covariate) > (HA × covariate)							
Cuneus		R	97	3.77	14	−70	26
(HA × covariate) > (AA × covariate)							
Insula		L	77	4.23	−38	−20	−2
Fusiform gyrus		R	32	3.20	30	−50	−18
Postcentral gyrus		R	46	2.96	56	−10	32

*Note.* Voxels = number of voxels per cluster; x, y, z = MNI coordinates of peak voxel; AA = anxious adolescents; HA = healthy adolescents; R = right side; L = left side; PFC = prefrontal cortex; *p* < .05, corrected

Group differences in the whole-brain regression analyses of PPI x sleep-amount identified four clusters (Table 4b). One cluster showed stronger correlation of right cuneus-hippocampus coupling x sleep-amount in AAs than in HAs. The opposite was found for the other three clusters located in the left insula, right postcentral gyrus, and right fusiform gyrus.

Further correlational analyses were conducted on hippocampus connectivity with left insula to determine the direction of the observed effects. Correlations were in opposite directions for the AA and the HA groups (Fig. 4b). AAs showed a positive correlation between sleep-amount and hippocampus-insula coupling. HAs showed effects in the opposite direction.

## Discussion

This is the first study to investigate the interaction of clinical anxiety and subjectively reported sleep amount/tiredness on emotion processing in adolescents. We predicted that the amount of sleep obtained over the 3 nights preceding the study would influence the neural processing of negative stimuli, and that this relationship would be different in anxious versus healthy adolescents. Of specific interest were the regions known to be consistently engaged in emotion processing, including hippocampus, amygdala, insula, and mPFC (Etkin et al., 2011; Hartley & Phelps, 2010; Milad et al., 2007; Ochsner et al., 2004; Phelps, 2004). Accounting for sleep amount in AAs and HAs, two key results emerged: (1) The effects of sleep amount on brain response to negative stimuli revealed significant group differences in four regions, including two a priori hypothesized regions, the dACC and hippocampus; (2) The effects of sleep amount on the dACC-seeded PPI connectivity with dmPFC (dACC–dmPFC) and on the hippocampus-seeded PPI connectivity with insula (hippocampus–insula) significantly differed between AAs and HAs. Subjective reports of tiredness, however, did not modulate neural responses to negative emotional faces.

The initial whole-brain finding of reduced vmPFC activation to negative faces in AAs relative to HAs is consistent with the wealth of evidence associating anxiety with reduced engagement of this region during regulation of negative affect in adult and adolescent samples (Arnsten & Rubia, 2012; Diekhof et al., 2011; Etkin et al., 2011; Greenberg et al., 2013; Maier et al., 2012; Mcclure et al., 2007; Morgan et al., 1993; Quirk & Beer, 2006; Quirk et al., 2003; Schiller & Delgado, 2010). The vmPFC has been implicated in the control of negative emotion through inhibitory projections to subcortical limbic regions, particularly the amygdala (Etkin et al., 2011; Morgan et al., 1993; Quirk & Beer, 2006; Quirk et al., 2003; Shackman et al., 2011). Moreover, lesion studies in rodents have shown that this region is important in sleep regulation (Chang, Chen, Qiu, & Lu, 2014). Therefore, disruption in the vmPFC may contribute to both poor sleep patterns and deficits in emotion regulation, including anxiety (Chang et al., 2014), perhaps supporting the common association of sleep problems with anxiety disorders (Gregory & Sadeh, 2012; Reid et al., 2009; Tkachenko et al., 2014).

### Sleep amount on whole-brain responses to negative faces

With respect to the effects of sleep amount on whole-brain activation to negative faces, the effects of reduced sleep amount on emotion-related responsivity were expected to be amplified in AAs relative to HAs. Findings in AAs were more complex than a simple exaggeration of the effects of low sleep amount in HAs. Two regions involved in emotion processing, dACC and hippocampus, were affected by sleep amount differently in AAs and HAs. Both regions showed similar patterns of association with sleep amount, that is, positive association in AAs (less sleep, less activation) but negative in HAs (less sleep, more activation).

### The dACC

The dACC has been implicated in emotion evaluation and integration with autonomic signals, generating appropriate behavioral responses (Etkin, 2009). In line with its role in cognitive processes (e.g., Bush, Luu, & Posner, 2000; Ridderinkhof, Ullsperger, Crone, & Nieuwenhuis, 2004), the dACC monitors emotional information and activates appropriate control processes (Egner, Etkin, Gale, & Hirsch, 2008). The present finding fits with the established association of higher emotional reactivity with sleep deficits in healthy subjects (Chorney et al., 2008; van Der Helm & Walker, 2009), as well as relationships between poor sleep quality and dACC activation (Klumpp et al., 2017; Minkel et al., 2012). In contrast, the opposite direction of sleep–BOLD activation association in anxious patients is puzzling. The less sleep anxious adolescents reported, the weaker was their dACC response to negative stimuli. A possible interpretation is that this reduction in responsivity might reflect an overall neural hyperexcitability, not specific to negative stimuli, in anxiety disorders. Accordingly, while the contrast between negative versus neutral responses would be reduced, independent activation in each state would be heightened. Preliminary work with magnetoencephalography in adults shows that this is a possibility, based on the observation of overall decreased alpha wave activity in anxious versus healthy adults, both during threat induction and safe conditions (Balderston et al., 2017).

Another interpretation can be considered based on the sleep-related modulation of dACC functional PPI connectivity. Indeed, PPI analysis revealed that the negative connectivity of dACC with dmPFC was influenced by sleep differently in AAs versus HAs. Reduced sleep amount corresponded with weakened positive dACC–dmPFC PPI connectivity in HAs, but with strengthened connectivity in AAs. This cluster, located within Brodmann area 8 (cluster peak MNI (x, y, z) = 16, 22, 46), is part of the dorsal attention network (Ahn et al., 2014), and, in concert with the dACC, has been implicated in threat appraisal and response to uncertainty (Etkin et al., 2011). Specifically, the dmPFC is a key node of the emotion-regulation network (Ochsner, Silvers, & Buhle, 2012; Pessoa, 2009). The most parsimonious interpretation of this finding is that sleep amount modulates the efficiency of dmPFC–dACC function. Accordingly, in healthy subjects, more sleep would be associated with higher efficiency of the emotion control system, through a strengthening of dmPFC–dACC connectivity and reinforcement of top-down emotion regulation. In anxiety disorders, reduced sleep-amount would lead to the failure to recruit control regions (i.e., dACC), leading to an ineffective strengthening of dmPFC–dACC connectivity.

### Hippocampus

The hippocampus has been consistently implicated in both anxiety disorders and sleep disorders. In anxiety disorders, the hippocampus plays a role in fear memory and contextual anxiety (Shin & Liberzon, 2009). In sleep disorders, the hippocampus has been associated with memory impairment and exhibits atrophy in chronic sleep deprivation (Guzmán-Marín et al., 2003; Mcewen, 2006). Despite the prominence of sleep perturbations in anxiety disorders and the hippocampus being a key region in both anxiety and sleep problems, this region has not been examined specifically with regard to its relationship in the link between clinical anxiety and sleep problems. One study has reported that enhanced hippocampus activation to fearful stimuli was associated with decreased sleep in healthy individuals (Motomura et al., 2013). In the present study, the same relationship was found in HAs. However, this relationship was opposite in AAs. In patients, the less sleep that was accumulated, the less activation to negative stimuli was seen in the hippocampus. A similar interpretation to that discussed above could be proposed; less sleep may be associated with potentiation of the already-present brain hyperexcitability in anxiety disorders, resulting in the decrease of differential activation between neutral and negative stimuli. The lack of an absolute measure of brain activity of the BOLD signal mitigates the possibility to test this speculation. However, through an orthogonal manipulation of sleep amount and anxiety, it may be possible to shed light on this relationship.

The hippocampus-seeded PPI analysis also showed an abnormal relationship of hippocampal connectivity and sleep amount in anxious patients. Sleep amount was found to modulate the connectivity of hippocampus with insula differently in AAs and HAs. Shorter sleep amount was associated with reduced hippocampus–insula connectivity in HAs, but with enhanced hippocampus–insula connectivity in AAs. Recently, a meta-analysis of both functional and structural neuroimaging studies implicated hippocampus and insula hypoactivation in patients with poor sleep (obstructive sleep apnea; OSA; Tahmasian et al., 2016). However, analogies of findings from meta-analyses of neuroimaging studies to the present work should be made with great caution because of the differences in the population and sleep factor (e.g., adults with a sleep disorder vs. adolescents with variable but typical sleep amount). Nonetheless, this meta-analysis highlights the same two regions, dACC and hippocampus, as being implicated in sleep abnormalities. With this caveat in mind, it is interesting to note that the hypoactivation of the hippocampus in OSA patients is reminiscent of that in the AA group but not the HA group observed in the present investigation. Although comorbid psychiatric disorders were not reported in these OSA patients, it is possible that many of them suffered from anxiety, a common condition in OSA.

Stronger connectivity between insula and hippocampus with shorter sleep amount in AAs might underlie hyperactive processing of negative stimuli generated by a hyperresponsive insula, modulating hippocampal encoding of negative stimuli (Phelps, 2004). The insula is implicated in anxiety (Etkin & Wager, 2007; Goodkind et al., 2015; Hattingh et al., 2013; Kalisch & Gerlicher, 2014; Stein et al., 2007) and has been shown to work in concert with the hippocampus in encoding negative, but not positive, faces (Tsukiura, Shigemune, Nouchi, Kambara, & Kawashima, 2013). In contrast, the weakening of this connectivity in HAs with poor sleep suggests a disorganization of the coding of negative stimuli, associated with a dysregulation of insula–hippocampus coupling.

Taken together, it is also useful to consider the findings in the framework of fear-related networks more generally. In the context of fear processing, two distinct networks have been proposed: a more dorsal fear-expression network, comprising the dACC and insula, and a more ventral fear-extinction network, comprising the vmPFC and the hippocampus (Milad & Quirk, 2012; Milad & Rauch, 2012). The implication of these regions in the present findings suggests that perhaps sleep impacts both of these opposing networks in the context of anxiety, and the dissociation of these effects indeed merits further investigation.

Finally, the absence of modulation by sleep amount of amygdala response to negative faces was somewhat surprising. While previously shown to be involved in various facets of the face-attention task (e.g., Guyer, Monk, et al., 2008; Mcclure et al., 2007), amygdala response to negative faces did not differ between AAs and HAs, nor was it modulated by sleep amount. One possibility is that our BOLD contrast pooled together four different attention states (passive viewing, attention to subjective feeling, attention to physical feature, attention to facial emotion) while viewing negative faces. The pooling of the attention states in the present analysis was motivated by the following rationale: first, the use of all task stimuli in the analysis maximized the statistical power to detect sleep effects; second, we had no a priori hypotheses regarding the potential effects of sleep on the different attention types. However, pooling data across attention states might have prevented us from detecting potential findings in the amygdala, as this region has been shown to respond distinctly under different attention constraints and emotion types (Guyer, Monk, et al., 2008).

### Limitations

A number of limitations should be considered. First, the sample size was relatively small. Although smaller sample sizes are adequate for detecting effects in fMRI (Thirion et al., 2007), larger samples are needed to detect associations between behavioral measures and BOLD signal changes. This issue might explain why we did not observe group differences in performance or sleep variables, or behavioral–BOLD response associations. Moreover, the anxious group reported less sleep amount on average, with wider standard deviations and range, suggesting that some participants in this group may have had more significant sleep problems despite no between-group differences. However, ultimately, the fact that AAs and HAs did not differ on sleep variables supports the argument of differential effects of sleep specifically on brain function between these groups, while these effects may not necessarily manifest behaviorally. Future work should aim to replicate the present findings with larger samples to increase statistical power. In addition, the use of self-report to index sleep may have influenced the results. For example, work has shown that self-reported sleep duration correlates moderately (Lauderdale, Knutson, Yan, Liu, & Rathouz, 2008) or even poorly (Regestein et al., 2004) with objectively measured sleep and that this relationship may be affected by the presence of psychological difficulties, including depression. Future studies may inform this issue. For example, indexing questions from the diagnostic interview that specifically pertain to sleep problems may be an additional informative variable by which to distinguish groups as a function of sleep problems (e.g., sleep-disturbance-associated vs. non-sleep-disturbance-associated), as it is possible that some forms of anxiety, such as GAD, more commonly co-occur with sleep problems compared to, for example, social anxiety. Similarly, six of 14 anxious patients had comorbid depression, which may have further confounded results, although comorbidity between these two diagnoses is relatively common and covarying for CDI scores did not affect results. Additionally, this is an observational study of the effects of sleep on emotion processing in function of clinical anxiety. Compared to other measures, such as the Pittsburgh Global Sleep Quality Index (PSQI; Backhaus, Junghanns, Broocks, Riemann, & Hohagen, 2002), the sleep measure in this study has not been empirically validated in prior work. While the questionnaire used in this study has face validity and other studies have used self-report measures to investigate neural correlates of subjectively reported sleep amount and tiredness (Killgore, 2013; Klumpp et al., 2017; Reidy et al., 2016), the questions rely on retrospective self-report up to 3 days before testing, which may have impacted results based on self-report bias that has been previously associated with psychological difficulties (Lauderdale et al., 2008). Moreover, sleep quality, onset, or latency are sleep measures that may be more informative indices than sleep amount measured in this study. Therefore, future studies should aim to assess sleep patterns using more traditionally validated subjective self-report measures, such as sleep diaries, as well as measures such as the PSQI capturing qualitative variables, or using more real-time, objective methods, such actigraphy. Moreover, the presence of wake time periods during sleep should be taken into account, as sleep interruptions are common in anxiety (Lee, 2011). In addition, this study used typical sleep patterns and does not inform research on sleep disorders. However, this retrospective, self-report study assessing sleep amount and tiredness provides preliminary findings that can guide future work on sleep, brain function, and anxiety. Finally, the absence of group differences in reported tiredness might be related to factors other than sleep amount, as shown by the lack of correlation between sleep amount and tiredness in HAs. For example, the feeling of tiredness is a subjective, less reliable measure than the number of hours slept, sleep onset latency, or nighttime wake times, particularly in anxiety patients.

## Conclusion

This is the first study to provide preliminary evidence showing that anxiety may moderate how sleep amount affects the neural processing of negative emotions depicted through facial expressions. These preliminary findings support the possibility that sleep has a distinct effect in clinical anxiety on the neural circuitry of fear and emotion processing. These initial findings should be examined in larger studies with more robust sleep measures to further inform the neural mechanisms linking sleep problems with anxiety (Hettema, Neale, & Kendler, 2001). Insights into these mechanisms might be used in treatment development for anxiety and sleep dysfunction.

## Electronic Supplementary Material

ESM 1 (DOCX 279 kb).
