# Supplementary material for: Sleep-amount differentially affects fear-processing neural circuitry in pediatric anxiety: A preliminary fMRI investigation
Source: Cogn Affect Behav Neurosci. 2017 Sep 14;17(6):1098–113. doi: 10.3758/s13415-017-0535-7 (PMC5709437; doi:10.3758/s13415-017-0535-7)
Supplement: Supplementary file 1 [file 13415_2017_535_MOESM1_ESM.docx]

Supplementary Material

Sleep-amount differentially affects fear-processing neural circuitry in pediatric anxiety: A preliminary fMRI investigation

Carlisi, Christina O, PhD, Hilbert, Kevin, Guyer, Amanda E, PhD, Ernst, Monique, MD, PhD

Supplementary Results

Sleep measures

Supplementary Figure S1. 95% Credible Intervals, Bayesian independent samples t-tests

Task performance

Mean reaction times (MRT) by face-type are presented in Table S1. Repeated measures ANOVA showed no main effect of group on MRT (BF10=0.33). Likewise, no significant group differences were found for MRT difference in rating negative faces, i.e., [fear+angry faces]–neutral faces. This lack of group effect on reaction time (RT) measures is consistent with previous research using similar versions of this task ([Mcclure et al., 2007](#_ENREF_2); [Guyer et al., 2008](#_ENREF_1)). Across groups, sleep-amount or tiredness did not correlate with RT on any trial type. 

Supplementary Table S1. Task Performance Data

|  | Healthy Adolescents | Anxious Adolescents |
| --- | --- | --- |
| Face type (MRT in ms) | Mean | SD | Mean | SD |
| Angry | 1821.00 | 339.46 | 1661.26 | 475.67 |
| Fearful | 1804.76 | 291.87 | 1594.53 | 502.30 |
| Angry + Fearful | 1812.88 | 303.28 | 1627.89 | 479.01 |
| Neutral | 1679.84 | 300.33 | 1569.65 | 484.71 |
| (Angry+Fearful)-Neutral | 133.04 | 218.00 | 58.24 | 279.05 |

Abbreviations: MRT: mean reaction time; SD: standard deviation. 

Supplementary Figure S2. Comparison of whole-brain activation to negative>neutral faces in anxious patients relative to healthy controls

(A) AA > HA					     (B) HA > AA 

Fig S2. Axial brain slices depicting whole-brain activation differences between (A) anxious adolescents (AA) relative to healthy adolescents (HA) and (B) healthy adolescents (HA) relative to anxious adolescents (AA) when viewing negative faces (versus neutral faces). The right side of the image corresponds to the right side of the brain, p<0.05 corrected.

Supplementary Figure S3. Within- and across-group whole-brain activation maps for main effects of sleep-amount during negative > neutral faces

(A) Healthy Adolescents					    (B) Anxious Adolescents

  (C) All Participants

  

Fig S3. Axial brain slices depicting the main effect of sleep-amount on brain activation during the viewing of negative (relative to neutral) faces (A) in the group of healthy adolescents (B) in the group of anxious adolescents and (C) across all participants. The right side of the image corresponds to the right side of the brain, p<0.005 uncorrected.

References

Guyer, A. E., Monk, C. S., McClure-Tone, E. B., Nelson, E. E., Roberson-Nay, R., Adler, A. D., Fromm, S. J., Leibenluft, E., Pine, D. S., & Ernst, M. (2008). A Developmental Examination of Amygdala Response to Facial Expressions. Journal of Cognitive Neuroscience, 20(9), 1565-1582. doi: 10.1162/jocn.2008.20114

McClure, E. B., Monk, C. S., Nelson, E. E., Parrish, J. M., Adler, A., Blair, R. J., Fromm, S., Charney, D. S., Leibenluft, E., Ernst, M., & Pine, D. S. (2007). Abnormal Attention Modulation of Fear Circuit Function in Pediatric Generalized Anxiety Disorder. Archives of General Psychiatry, 64(1), 97-106.
